# A portable electrical impedance tomography based pressure mapping sensor and force localisation validation system

**DOI:** 10.1016/j.ohx.2025.e00628

**Published:** 2025-02-12

**Authors:** Richie Ellingham, Lui Holder-Pearson, Chris Pretty, Tim Giffney

**Affiliations:** a Mechanical and Mechatronic Engineering Department, Canterbury University, Ilam, Christchurch, Canterbury, 8041, New Zealand; b Electrical and Electronics Engineering Department, Canterbury University, Ilam, Christchurch, Canterbury, 8041, New Zealand

**Keywords:** Soft sensor, Pressure mapping, Artificial skin, Portable, Electrical impedance tomography, Research and development kit

## Abstract

This work presents portable, low-cost hardware for pressure mapping using EIT-based soft sensors. An important part of developing these EIT-based pressure sensors is the sensor characterisation. Therefore, this work also provides the design of a system for characterising and validating the spatial, pressure, and temporal performance of different soft sensor material domains. The system is capable of driving soft EIT-based sensors using a range of sensing materials, shapes, and configurations. The hardware allows for the wireless transmission of EIT data to a remote device. A data capture frame rate of 12.7 Hz allows for the analysis of dynamic events. The maximum current drive voltage is ±22 V and a voltage read resolution of ±0.3μV allowing for a range of sensing domain sizes, thicknesses, and materials. A Cartesian force applicator device has been developed for the automated pressure mapping sensor characterisation which can apply and sense loads from 0 to 100 N with a resolution of ±50 mN at rates of 0 - 800 mm/min. Loads can be applied with an error of ±0.01 mm. A standardised method has been provided for researchers to experiment with a range of different sensing domain materials and shapes. The system described in this work is suitable for both research and practical applications, making it a valuable tool for advancing the field of EIT-based soft pressure mapping sensor technology.

## Graphical abstract

**Figure:**
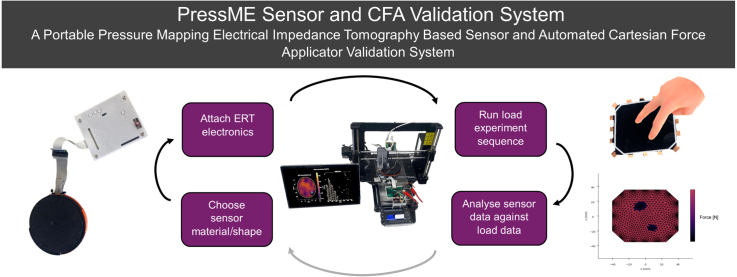

## Specifications table

**Table:** 

Hardware name	EIT Pressure Mapping Device and Calibration System

Subject area	Engineering and material science

Hardware type	• Imaging tools • Measuring physical properties and in-lab sensors • Mechanical engineering and materials science

Closest commercial analogue	No commercial analogue is available.

Open source licence	General Public License (GPL)

Cost of hardware	ERT sensor device : USD$148 Cartesian force applicator device : USD$1046 (incl. Prusa MK3s 3D printer - USD$899)

Source file repository	http://doi.org/10.5281/zenodo.14648206

## 1 Hardware in context

Electrical impedance tomography (EIT) is an imaging technique used to map impedance/resistance throughout a material domain using multiple boundary electrodes. The boundary electrodes inject current through the domain, allowing impedance mapping measurements to be non-invasively determined. EIT is most commonly used for thorax imaging for clinical respiratory analysis; however, this same method can be used for a multitude of applications with conductive bodies to map changes in impedance/resistance. Commercial devices that perform EIT, or the DC equivalent electrical resistivity tomography (ERT), exist in large-form factors such as Pulmovista 500 (Draeger, Luebeck, Germany), EIT16/32 (Sciospec Scientific Instruments GmbH, Leipzig, Germany), LuMon (Sentec, Lincoln, USA), Zeta (Zonge International, Tucson, USA), WGMD-4 (WTS Geophysical, Wanchai, Hong Kong). All of these options are application-specific for biomedical and geophysical applications. Several research papers [1], [2], [3], [4], [5], [6], [7], [8], [9], [10] have described similar, but smaller, versions of the commercial products mentioned. In this work the same physics principles used in bio-medical and geophysical sensing are used to map localised compressive loads on a soft piezoresistive material. Pressure mapping is widely used for many applications including sports equipment grip analysis, foot pressure in gait analysis, in production line part alignment, hospital patient bed and chair pressure minimisation, headphone pressure analysis, amongst many others. These applications are useful for increasing quality of life, optimising sporting performance, object detection, and production efficiency optimisation.

A core limiting factor of current soft pressure sensor technology is the lack of customisability in sensor size, shape, sensing domain material softness, and sensing material composition [11], [12], [13], [14]. Pressure sensors are often given in a rectangular format because of the arrays of wiring and sensing elements required within the sensing domain. EIT-based pressure mapping sensors are not constrained by wires or complex patterning within the sensing domain area. EIT-based pressure sensors can have their sensing domain configured in various 2D shapes depending on the material and fabrication methods used.

Note that throughout this work the term EIT is used when referring to the process of reconstructing a conductivity image from voltage data, whereas ERT is used when referring to the process of capturing voltage data from a sensing domain. This work creates an ERT device not an EIT device to reduce complexity by only using DC-based measurements. To advance the research of this soft pressure mapping platform technology, we require a system that is lost cost, open source, easy to use, portable, and sufficiently flexible to test a range of different sensing domain materials. The system developed in this work is for the research and development of EIT-based pressure mapping sensors.

The hardware of our system has two key components, a circuit for gathering raw ERT data and a Cartesian force applicator (CFA) machine for characterising an ERT-based pressure mapping sensor. The system characterises the sensor and can be used for validating the spatial, pressure, and temporal performance for different piezoresistive sensor material domains. The CFA allows for repeatable experiments and quantifiable data for different sensor configurations.

## 2 Hardware description

**Fig. 1:**
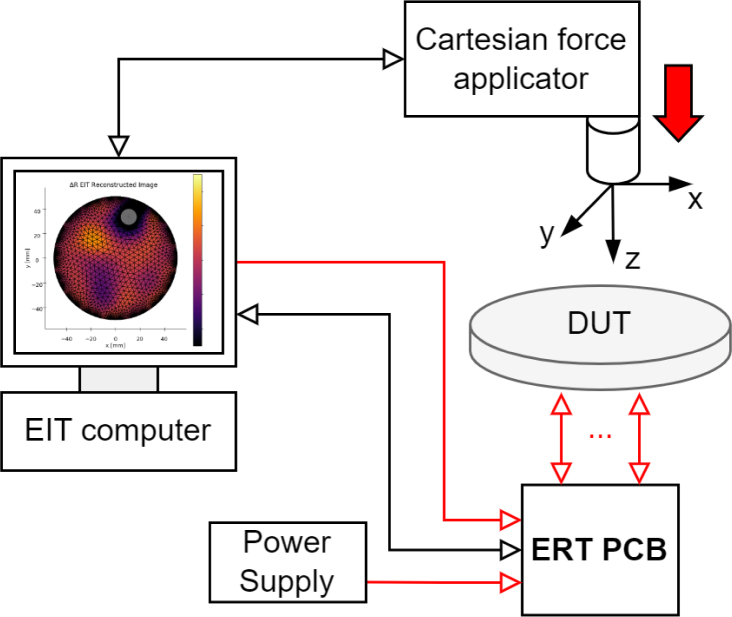
System architecture of the ERT sensor and CFA setup. The large red arrow shows the direction of the force applicator compression onto the sensing domain under test (DUT). Analogue/power signals are shown with red arrows and digital signals with black arrows. (For interpretation of the references to colour in this figure legend, the reader is referred to the web version of this article.)

This work describes the design and creation ERT-based pressure mapping sensor toolbox that reliably and repetitively allows for ERT-based pressure mapping and quantification of the sensor performance. The overall system is simple to construct, easy to operate, and is split into two main parts: the ERT sensor and the CFA device as shown in Fig. 1.

**Fig. 2:**
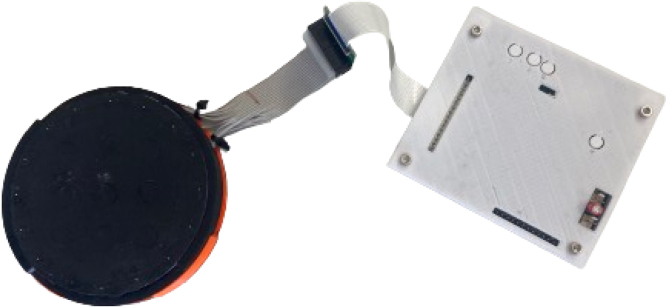
A soft sensor domain connected to the ERT sensor electronics.

The ERT sensor consists of an ERT sensor circuit and the sensing domain under test (DUT). The ERT circuit drives the EIT measurements through the soft sensing domain material. The EIT circuit designed is small (79 × 94 × 12 mm) for potential use in space-constrained mobile applications as seen in Fig. 2. The system has a programmable current source which can drive up to 50 mA of constant current. The voltage measurement circuit has an ADC resolution of 0.3 μV, ensuring that the small signals generated by small localised loads can be detected. The sensing domain demonstrated in this work is a soft piezoresistive composite made from carbon black (CB) powder and silicone rubber with 16 boundary electrodes made from gold pins and copper tape, as seen in Fig. 6.

**Fig. 3:**
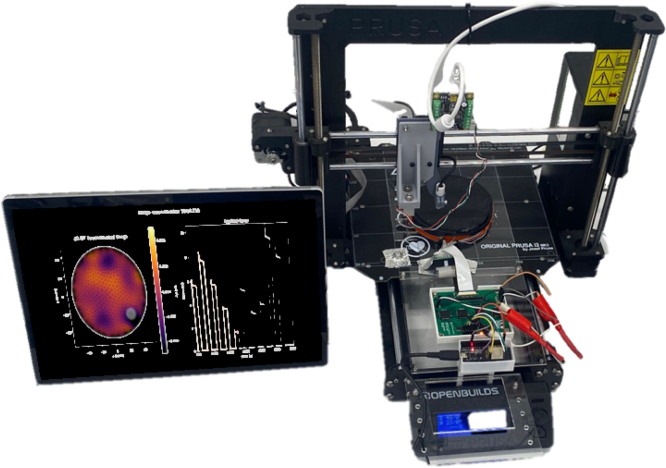
Cartesian force applicator setup with an ERT circuit and EIT reconstruction computer.

To ensure that the sensor can accurately locate pressure points and their magnitude, the CFA device described in this work is used to apply compressive forces at specifically programmed locations. The CFA test bed allows for loads within a 220 × 180 mm area and a picture of the CFA mid-experiment is given in Fig. 3.

Previous research groups have developed EIT hardware for pressure mapping sensors [1], [15], [16], [17], [18]; however, a complete open-source system including validation hardware has not yet been published to the best of the authors’ knowledge.

To move the field of EIT-based soft pressure mapping forward, there is a need to optimise materials for qualities such as pressure sensitivity, homogeneity, electrode connection reliability, and dynamic viscoelastic properties. This CFA automates the testing process with easily changeable spatio-temporal parameters, such as strain magnitude, strain rate, and strain profile. This system helps standardise the analysis of the pressure mapping by allowing for the EIT reconstructed resistance images to be compared with stress and strain data form the sensing domain material.

The hope for the hardware and software given in this paper is that it will provide a standardised platform for future researchers to use to further quantify the utility of other sensing materials, and their compare their performance metrics with standard loading test procedures, as done in our previous work [19].

The ERT sensor and force applicator hardware could be utilised in further research for:

• 2D piezoresistive material analysis
• Pressure mapping device characterisation and performance
  – Spatio-temporal performance
  – Dynamic stress sensing performance
  – Piezoresistivity
• Development for real-world applications
  – Robotic skin integration
  – Sports sensing
  – Prosthetic limbs

## 3 Design files summary

The ERT pressure mapping sensor contains a PCB assembly, housing, and wiring. The CFA design includes a selection of off-the-shelf parts as well as custom designed mechanical CAD parts. Table 1 contains all of the custom designed parts from the system as well as the software for both the ERT sensor and CFA devices. In this work the mechanical (CAD) files were generated using Solidworks and the electrical (ECAD) files were generated using KiCAD. Programs designed for this system were mainly written in Python and C.

**Table 1:** Summary of all design files.

Designed parts	File types	Open source licence	Location of the file
ERT pressure mapping sensor			
PCB design	elec CAD (.kicad_sch, .kicad_pcb, .kicad_pro)	GPL	10.5281/zenodo.11520112 /ERT_sensor/elec_CAD/

3D printed housing	mech CAD parts (.stl, .sldprt)	GPL	10.5281/zenodo.11520112 /ERT_sensor/mech_CAD/

PCB firmware	embedded firmware (.c)	GPL	10.5281/zenodo.11520112 /ERT_sensor/firmware/

ert_sensor_bom.csv	bill of materials	GPL	10.5281/zenodo.11520112 /ERT_sensor/

Cartesian force applicator			

Fabricated parts	mech CAD parts (.stl, .sldprt)	GPL	10.5281/zenodo.11520112 /CFA/mech_CAD/

Testing software	data capture/ processing (.py)	GPL	10.5281/zenodo.11520112 /CFA/software/

cfa_bom.csv	bill of materials	GPL	10.5281/zenodo.11520112 /CFA/

### 3.1 ERT pressure mapping sensor file descriptions

**PCB design** - KiCAD project files including the electrical schematics and PCB layout for the ERT sensor circuit.

**3D printed housing** - CAD files for 3D printed sensor enclosure and a sensor domain holder example.

**PCB firmware** - Firmware for the ERT data capture driving the electrode drive pattern through multiplexer switching. The data captured is then streamed via serial to a separate reconstruction processor.

**ert_sensor_bom.csv** - Bill of materials for all parts and components in the ERT PCBA.

### 3.2 Cartesian force applicator file descriptions

**Fabricated parts** - CAD for 3d printed and laser cut parts for the modification of the 3D printer platform into a CFA.

**Testing software** - Software for simultaneous force, position, and ERT data acquisition and processing.

**cfa_bom.csv** - Bill of materials for all parts in the CFA system.

## 4 Bill of materials

Please refer to the two detailed bills of materials (BOMs) given in Table 1.

## 5 Build instructions

The build is separated into two parts. The first being the assembly of the ERT sensor electronics and housing. The second being the build of the CFA validation system, used to apply compressive forces to the sensor material domain. Text within square brackets refer to the part reference designators (e.g. [U1] is for the ESP32 module) in the BOMs.

### 5.1 ERT sensor

The manufacturing process of the PCB involves first sending the PCB gerber files to a PCB manufacturer. This work used JLC PCB with their default parameters for a 4 layer PCB.

**Fig. 4:**
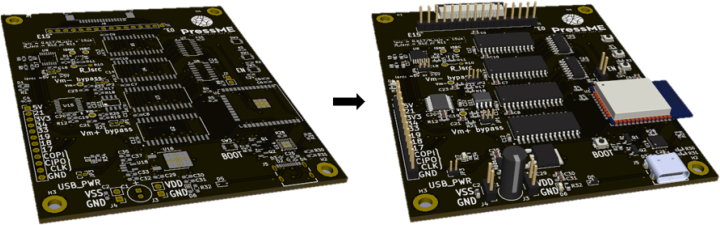
ERT PCB [PCB1] before and after electrical component population.

Next populate the PCBs with the SMD parts given in the BOM and place in a reflow oven. First complete the rear side then the top side to ensure components stick. Once all SMD parts have been soldered, solder all of the THT components as shown in Fig. 4. Finally attach the jumpers for the desired power mode, explained in Section 6.1.

To ensure simple protection against electrical shorts and low-level ingress protection (equivalent to IP20) 3D print an enclosure in PLA using the STL files given in the BOM, *ert_housing_top.stl* [PR1] and *ert_housing_base.stl* [PR2]. There are 4 threaded inserts [HW2] to mount the PCB securely in the 3D printed enclosure using four M3 14 mm bolts [HW1].

Attach the 16 way ribbon connector [W1] to the ERT electrodes. In this example there is a ribbon to IDC connector interface board [J8], which then connects to a custom built electrode pin to domain interface [DUT1, DUT2, PR3, PR4]. This electrode interface will vary based on the required sensing domain.

**Fig. 5:**
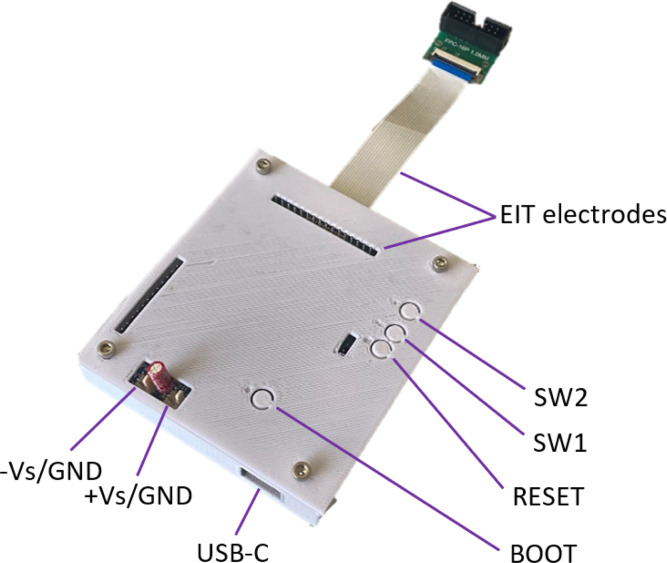
ERT sensor PCBA mounted in enclosure and attached electrode harness showing buttons and the main electrical connections.

The sensor device shown in Fig. 5 shows all of the connections and buttons necessary for the programming and operation of the sensor, as well as two optional buttons SW1 and SW2 for any other desired functions.

### 5.2 Sensing domain

The simple fabrication of a composite material with proven compatibility with EIT-based sensing is used as a reference is in this section. This work will utilise a material demonstrated in previous work [19]. This material optimises use for pressure sensing and has characteristics akin to human skin and muscle tissue including, a low Shore hardness of 5 A–25 A [20], [21], low viscoelasticity, high yield strength, low resistivity, high strain gauge factor, and low toxicity. Other sensor domain materials may be used for the sensor, such as soft conductive particle composites, conductive polymers, and hydrogels [1], [22], [23].

**Fig. 6:**
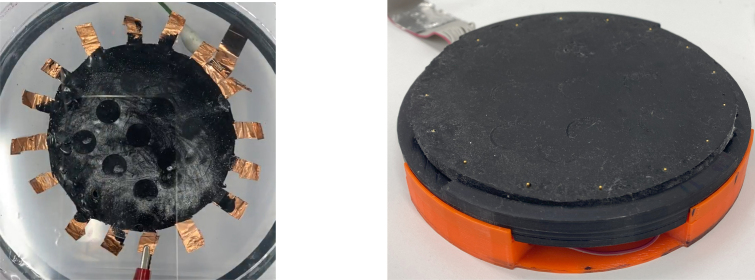
Left: An example of a CBSR sample with copper tape electrodes which has been integrated with a dielectric elastomer actuator setup [25]. Right: Example of a CBSR sensing domain with gold pin electrodes penetrating material surface around the sensing region boundary.

The sensing DUT demonstrated in this work was a composite comprised of XC 72R carbon black (CB) nanoparticles (Cabot, Alpharetta, USA) [DUT5] of 50 nm average diameter, dispersed in a two part Dragon Skin 10 NV silicone rubber (SR) matrix (SmoothOn, Macungie, USA) [DUT6]. The weight percentage (wt%) of CB to liquid silicone rubber which resulted in near optimal piezoresistive characteristics was found to be between 8%–10%. To ensure homogeneous CB particle dispersion and mitigate air bubble formation an ARV-310 vacuum planetary mixer (Thinky, Tokyo, Japan) was used to mix the CB particles through the liquid silicone matrix. Upon completion of mixing, the uncured composite was poured into a 100 mm diameter 4 mm thick disc mould. The curing of the composite was controlled by heating the newly-mixed material in the mould at 80 °C for 90 min. The domain samples used in this work and previous work [19], [24] had a diameter of 100 mm as shown in Fig. 6.

Researchers fabricating their own sensing domain for use with this system should follow three key requirements,

1. The size of the sensing domain must fit within a 220 × 180 × 160 mm volume (X × Y × Z) on the CFA test bed.
2. The bulk modulus of the domain material must be chosen such that the required loads applied to the domain must not exceed 100 N.
3. The inter-electrode resistance, $R_{int}$, must be low enough to not saturate the current source, $I_{src}$, given a power supply voltage, $V_{s}$, as shown in Eq. (1),
  (1) $$R_{int}<\alpha \frac{V_{s}}{I_{src}}$$

where $R_{int}$ is the resistance values between every configuration of the current drive electrodes during an EIT capture cycle and α is the factor of safety for any electrode movement or incidental increase of $R_{int}$ during experimentation.

### 5.3 Cartesian force applicator

To test the spatial and force resolution of the ERT pressure mapping device, the CFA was designed using a Prusa MK3s 3D printer to provide a stable platform. First a functional Prusa MK3s 3D printer was acquired with its printing capabilities tested on several standard demo PLA prints to ensure the print head can move with the expected resolution in the X, Y, and Z directions. Standard benchmark tests and tuning for the printing platform can be found here and here [26], [27].

**Fig. 7:**
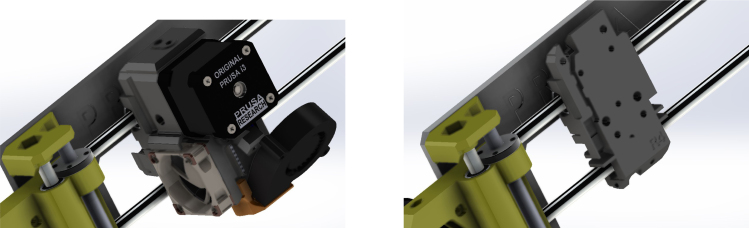
MK3s print head. Left: Original print head assembly. Right: Dismantled print head assembly [28].

**Fig. 8:**
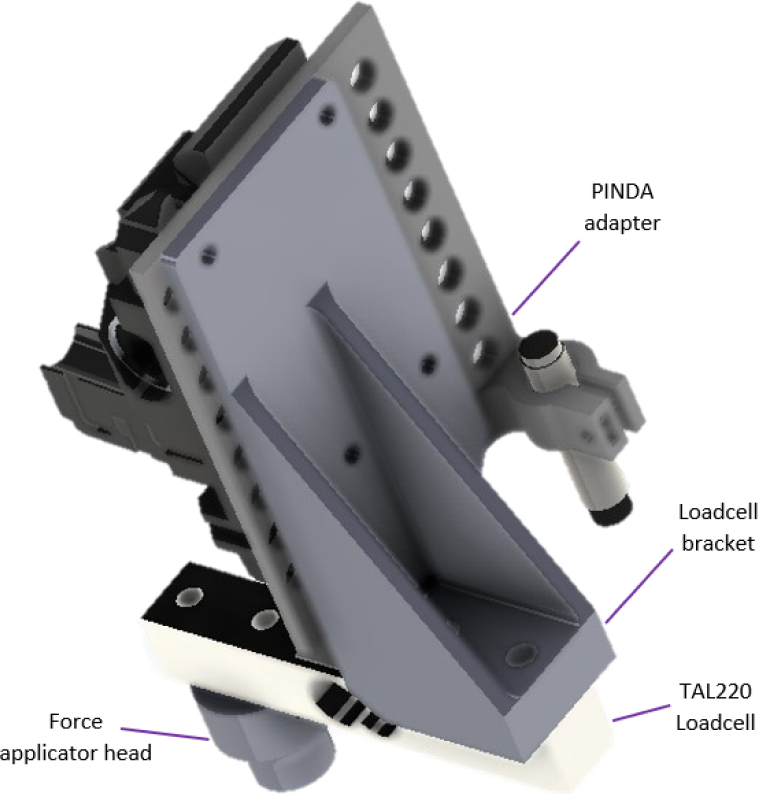
Force applicator head assembly.

Next the print head of the MK3s was dismantled, as shown in Fig. 7, leaving a flat surface to attach first the PINDA adapter [PR8] and the loadcell bracket [PR9], as shown in Fig. 8. Use M3 bolts to attach the loadcell bracket and PINDA adapter to the dismantled print head surface. See read the printer assembly manual for more detail on the part assembly [29]. Bolt the TAL220 10 kg loadcell [E3] onto the loadcell bracket using M5 bolts [HW4]. Bolt the force applicator head [PR5-7, HW5] onto the other end of the loadcell using M4 bolts [HW3]. A range of force applicator head shapes and sizes have been created to test the resolution of the sensor.

With the thermistor from the original printhead no longer required, a trimpot [RV1] is attached to the thermistor port on the printer’s control circuit PCBA [30]. While the 3D printer is turned on, the trimpot is manually adjusted until room temperature is reached (as shown on the 3D printer display) to avoid any future under/over temperature errors.

**Fig. 9:**
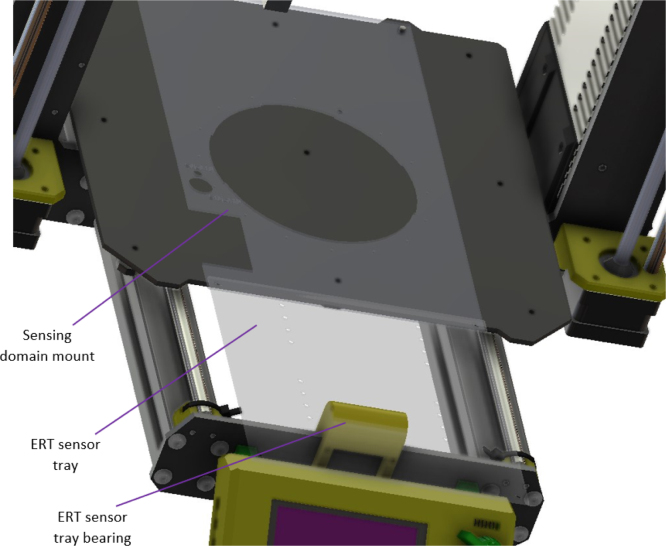
MK3s with original print bed tray removed and the ERT sensor trays added.

The print bed of the MK3s 3D printer is removed and replaced with a mount tray [LC1] for the sensing domain. The ERT tray bearing part [PR10] mounted and bolted [HW6] onto the frame of the MK3s as shown in Fig. 9. The ERT sensor tray [LC2] and the sensing domain mount tray are fixed together onto the print bed with two M3 bolts [HW6] clamping the trays to the edge of the print bed.

The firmware version used in this work was MK3s 3.9.0. Later versions may be compatible. Other 3D printer platforms with a similar gcode command set and a similar core firmware such as the Marlin firmware may also be used as a CFA. However, minor configuration changes in this work’s software and hardware may be required.

### 5.4 Design decisions

This section outlines and justifies some of the important design decisions made for both the ERT sensor and the CFA device.

**Fig. 10:**
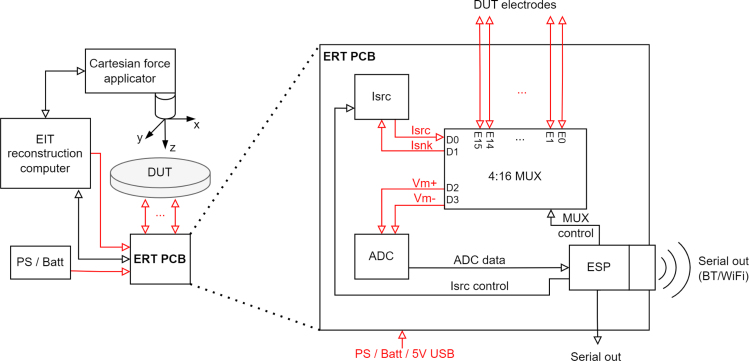
System architecture of ERT sensor and CFA setup (left) and the key internal electrical signals of the ERT circuit (right). With analogue/power signals shown with red arrows and digital signals with black arrows.

#### 5.4.1 EIT cycle and load sequence

**Fig. 11:**
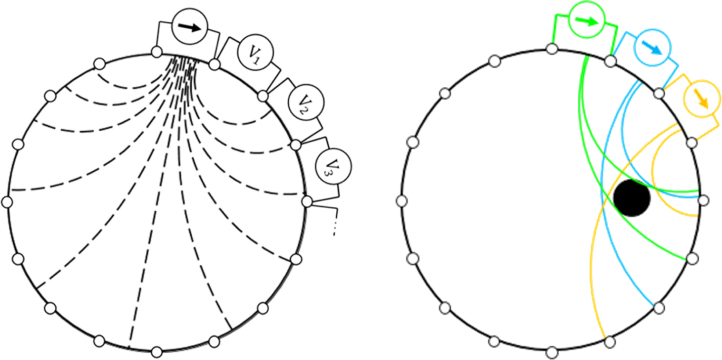
EIT adjacent drive pattern sequence. Left: Showing the voltage measurements being taking during a current injection. Right: Displaying that there are multiple current injections required when gathering reconstruction data.

While a sequence of compressive loads are applied by the CFA to the sensing domain, concurrently the ERT sensor circuit gathers data for EIT reconstruction using the circuit architecture given in Fig. 10. Previous research has shown the trade-offs with different EIT drive patterns [31], [32], [33]. This cyclic EIT data capture process follows a specific sequence,

1. A constant current is applied at adjacent electrode positions $E_{i}$ and $E_{i+1}$, where ‘i’ is the index of ‘n’ electrodes. These electrode positions are selected with the ‘MUX control’ line.
2. Sequentially 16 adjacent electrode voltage measurements are completed next (as exemplified in Fig. 11), again the electrode positions are selected with the ‘MUX control’ line.
3. Each raw voltage measurement is transmitted through ‘serial out’ to an ‘EIT reconstruction computer’.
4. The next current injection electrode position is selected (as exemplified in Fig. 11), i.e. i = i + 1 and return to step 2 unless i = n.
5. Once all 16 current injection positions are completed, 256 voltages are measured giving enough voltage data for one reconstruction frame. Each reconstruction frame can be then fed into an EIT reconstruction algorithm. Reconstruction frames are collected for the duration of the experiment.

Multiplexing of the voltage measurements was chosen instead of the alternative option of simultaneous voltage measurement, to maintain a low-cost circuit. The simultaneous voltage measurement solution involves 16 separate ADCs, one for each electrode. A DC current source was chosen as opposed to an AC source to simplify analysis and lower device cost. Although it has been shown that there are certain AC frequencies that are sensitive to a window of stress/strain values [34], [35], [36].

#### 5.4.2 Small signal measurement

The range of voltages required for recreating minute changes in resistances of a sensing domain spans several orders of magnitudes. Therefore a high dynamic range, high resolution, low-noise voltage measurement system is required to capture this data.

**Fig. 12:**
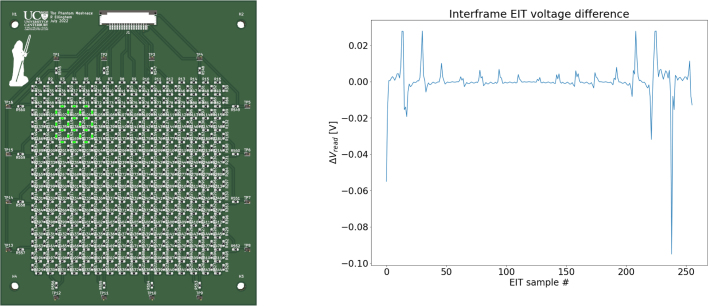
Left: A resistor mesh network for validating the ERT circuit, with the anomaly shown highlighted in green. Right: The difference between ERT raw voltage data from a homogeneous square 2.2 kΩ resistor mesh domain and the same domain with a 3.3 kΩ resistor mesh anomaly using PySpice. (For interpretation of the references to colour in this figure legend, the reader is referred to the web version of this article.)

To evaluate the performance of the system with a standardised testing domain a resistor mesh network was created. The mesh network was created to validate the expected resolution required for generating EIT reconstructions [24] for a variety of different resistances and resistance changes. The resistor mesh network provided a standardised platform with known resistor values and tolerances for the comparison a real and simulated resistor mesh network. The resistor mesh network was chosen to provide a range EIT voltage data comparable to that of the CBSR composite material demonstrated in Section 7. A guide to rapidly generating a square resistor mesh network, like the one shown in Fig. 12, is given in the connected repository in directory ‘*phantom_mesh*’.

A script was created to form a square resistor mesh network of various dimensions and various background resistance values. PySpice circuit simulator [37] was used to run a zero noise simulation on the system to show the expected difference in raw ERT voltage data, $\Delta V_{read}$, between a homogeneous resistor mesh and a resistor mesh with an anomalous blob as shown in Fig. 12. The maximum and minimum $\Delta V_{read}$ values shown in Fig. 12 and 94.994 mV and 53 μV respectively.

It is non-trivial to determine the exact resolution required for an EIT based pressure mapping sensor voltage measurements as it depends on the inherent noise of the domain, the force resolution required, the expected external noise, the drive current, the EIT reconstruction algorithm used, amongst other factors. This work uses a 24 bit ADC [U12] so that given an ideal noiseless representative domain the minimum voltage data shown in Fig. 12 will be an order of magnitude larger than the resolution of the ADC.

#### 5.4.3 Signal generation

The current source can drive a current, $I_{src}$ between 15μA and 50 mA and can be set as a programmable or fixed current source value. The $I_{src}$ value can be altered by changing the $R_{Isrc}$ [R7 or R8] value as shown in Eq. (2).

(2) $$I_{src}=\frac{0.617}{R_{Isrc}}+15\;\mu \text{A}$$

If a fixed current source is desired for the ERT circuit $R_{Isrc}$ sets the current source value based on Eq. (2). If the circuit is configured as a programmable current source, the digital potentiometer [U9] controlling the $R_{Isrc}$ value will a multiple of 39 Ω up to a maximum of 10 kΩ or a high impedance state. The possible programmable current source values are given in the isrc_lookup.xlsx file in the repository. If the resistivity of the domain is too high the current source supply will saturate to Vs. Ensure the domain resistivity is sufficiently low for this current source saturation not to occur within its expected range of use. A sufficiently high current value must pass through the domain to ensure low noise readings throughout the boundary electrodes on the domain. This noise predominantly occurs due to electrostatic effects in sensing domains. To ensure this current can be driven for the sensor domain configuration given in this work, a supply voltage, $\pm V_{s}$, of ±20 V should be used.

#### 5.4.4 Signal conditioning

**Fig. 13:**
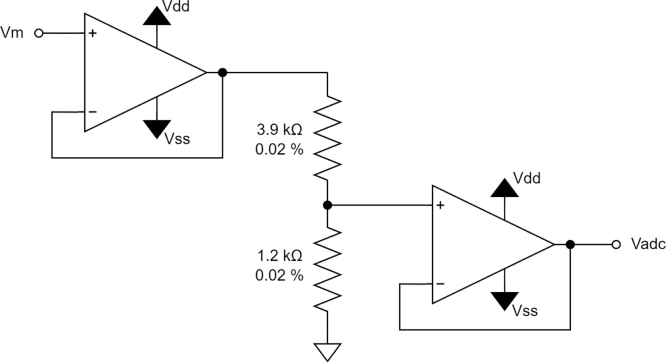
Measurement attenuation circuit.

When using the recommended supply voltage of ±20 V, an attenuation stage is required for the input into the ADC. This is done with an operational amplifier (opamp) voltage buffer-divider-buffer circuit as shown in Fig. 13. When using a single ended 5 V supply to drive the current source this opamp circuit can be bypassed using the jumpers shown in Fig. 18 as the attenuation is not required and the offset and noise due to the opamp circuit can be avoided. However, due to a lack of a negative $V_{ss}$, the multiplexer channel resistance will be higher and more variable as exemplified in Fig. 15.

To allow larger current signals to be driven through the domain a maximum voltage driving the current source of 20 V is used with an attenuation circuit consisting of two voltage buffers and a voltage divider. The attenuation circuit steps down the voltage with a nominal gain of 0.24 ± 3%. The attenuation circuit is duplicated for both differential ADC inputs. This circuit is highly sensitive to any noise, DC offset, or component variation. To combat the sensitivity of this circuit the opamps used [U10, U11] have a low input bias current, and low input DC offset voltage, the resistors used have a low tolerance of ±0.02%.

**Fig. 14:**
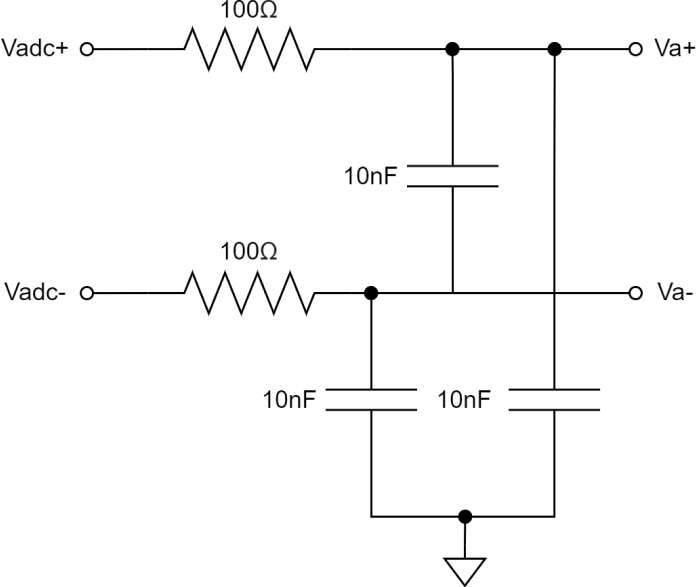
Passive low-pass filter circuit.

A passive low-pass filter has been placed between the opamp circuit and the ADC input to attenuate ADC input noise. The cutoff frequency for this filter has been set to a value of 1 MHz, with the resistance values shown in Fig. 14, allowing for sufficient settling time for the maximum potential ADC sample rate of 512 kSPS.

#### 5.4.5 Switching circuit

A 4:16 multiplexing circuit allows for a range of EIT switching drive patterns for ERT data acquisition. A multiplexer with a low drain–source on-resistance characteristic for smaller drain voltages has been chosen as the majority of the voltage readings being read through the multiplexer will be nearer to zero than ±Vs.

**Fig. 15:**
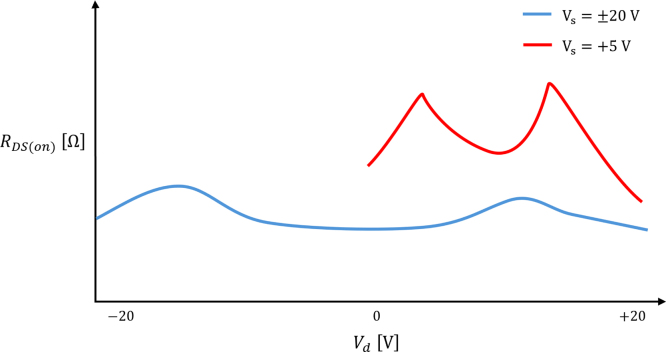
$R_{DS\left(on\right)}$ characteristic diagram for a typical multiplexer analogue channel for a dual and single-ended power supply [38].

Any variation in the $R_{DS\left(on\right)}$ value as a function of $V_{d}$ or from channel-to-channel will add to the offset noise read by the ADC lowering the resolution of the ERT pressure sensor. This low $R_{DS\left(on\right)}$ variation can be seen in Fig. 15. The multiplexer used can switch analogue voltage up to ±22 V with a switching time of 200 ns [38].

#### 5.4.6 Force measurement

**Fig. 16:**
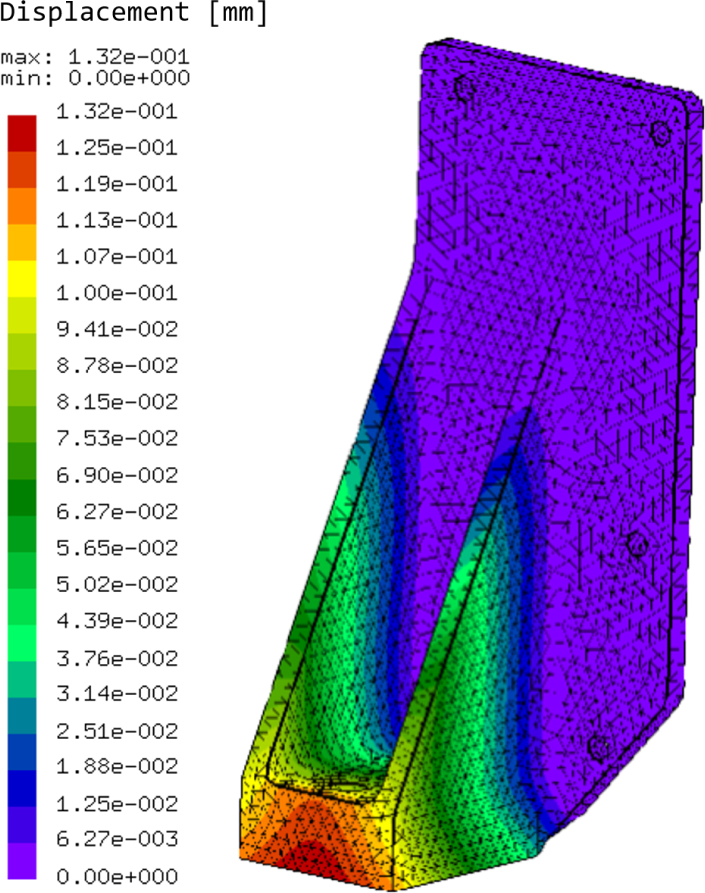
Static load analysis of the loadcell bracket part [PR9] of maximum allowable load of 100 N showing the magnitude of displacement.

The CFA system is designed to be used with soft piezoresistive sensing domains. Hence, the force applicator head must be significantly more rigid than the sensing domain being tested to ensure low strain error. To validate the force applicator components’ deformability a static load FEA simulation was completed on the loadcell bracket [PR9] with the maximum load expected used as 100 N. The material of the bracket was PLA with orthotropic material properties. The static simulation used the orthotropic 3D printed PLA properties given by Sosa-Vivas et al. [39] with Caculix FEM solver [40]. As shown in Fig. 16 the maximum predicted displacement of an FEM element within the loadcell part was 0.13 mm. If using a sufficiently soft domain and small force applicator this maximum displacement has little effect on the data processed, however this may cause significant error within harder domains and/or larger force applicators. Although the device can operate at 100 N, device operation is recommended below 50 N to decrease the strain error due to force applicator deformation.

The TAL220 loadcell used was chosen for the sensing domain material due to the force range, resolution, and high availability. The CBSR material had an elastic modulus of 100 kPa [19] so that range of strain measurements from 0%–50% could be accurately recorded with the given loadcell. The minimum force is on the limit of what can be detected as the TAL220 is rated for ±50 mN resolution [41] across its operating load and temperature range. However, the noise of the loadcell was measured to be ±2.5 mN during several 15 min sequentially loaded compression experiments in a 22 ± 0.7 °C regulated room. Examples of experiment loading limits are given in Table 2, giving the extreme cases for testing minimum and maximum theoretical strain for 5 and 20 mm diameter force applicator heads on a lower and higher elastic modulus material respectively.

**Table 2:** An example comparison of two extreme load cases with two different soft materials and two different force applicator head sizes. The table shows the minimum and maximum theoretical strain limits for 5 mm and 20 mm diameter force applicators and their respective required forces.

Force [N]	Force applicator diameter [mm]	Force applicator area [${\mathrm{mm}}^{2}$]	Domain elastic modulus [kPa]	Theoretical strain [%]	Theoretical stress [kPa]
0.06	5	19.6	60	5.1	3.1
50.00	20	314.2	200	79.6	159.2

#### 5.4.7 Position control and measurement

**Fig. 17:**
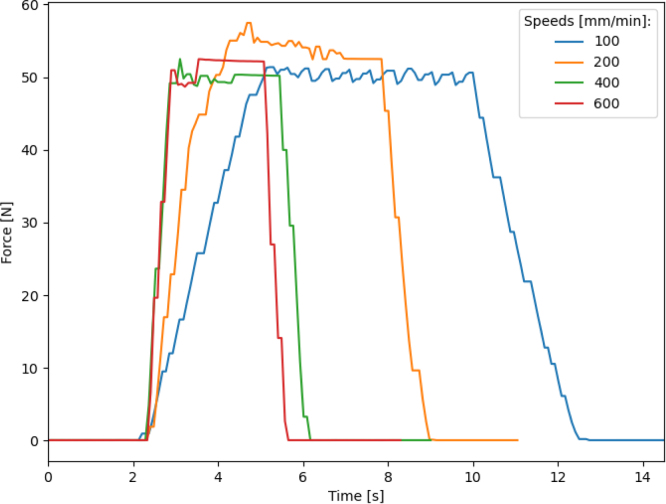
Comparison of different stepper motor speeds showing the stepper motor skipping and crash detection threshold from the TMC2009 drivers.

The Prusa MK3s 3D printer was used because of it is proven reliability as a 3D printer to move in x, y, and z axes with high resolution. The resolution of the force applicator location under without applying a load is 0.01 mm in each axis. Due to the open-loop nature of control of the stepper motors the resolution at high loads and high speeds may not be reliable. The Prusa MK3s comes with built-in crash detection software which determines when the stepper motor is skipping. Independent testing was completed to verify and quantify the force limits of the default crash detection, by driving the z-axis stepper motor into the print bed and measuring the force at which the motor skipped steps as seen in Fig. 17. This value is much lower than the over 100 N capabilities of the load cell.

#### 5.4.8 Sensing domain

The weight percentage of CB powder in an elastomer matrix, such as silicone rubber, to maintain desired mechanical and electrical properties can be tuned as shown in the characterisations completed by D’Asaro et al. and Shang et al. [42], [43]. The most desirable piezoresistive characteristics found in these works are near the inflection point of the conductivity versus CB weight percentage plot.

Because of the difference in fabrication processes and degree of dispersion generating variability in the percolation, an iterative trial and error approach using the starting point found in literature was used to get 8 wt % and 9 wt % values for CB in SR [42], [43]. Within this range the material was sufficiently conductive while maintaining mechanical strength through sufficient elastomeric cross-linking. Previous research indicates that there is a weight percentage at which the gauge-factor/piezoresistivity is at a maximum within a similar range used in this work [44], [45]. The CB particle dispersion can vary throughout a domain depending on various factors in the fabrication process including mixing technique, solvents used, silicone viscosity, particle size, particle agglomerations, amongst other factors [46], [47], [48], [49]. Dispersion of carbon black particles was ensured by using a relatively low viscosity silicone of 6,000 mPa.s and a centrifugal planetary mixer a method proven to give better dispersion than other traditional mixing techniques [50].

## 6 Operation instructions

To validate and characterise the ERT pressure mapping sensor the CFA is used to apply a sequence of loads to the material. The below sequence of required operations to complete this experiment includes:

1. ERT sensor power modes
2. ERT sensor programming
3. ERT sensing domain preparation
4. Load application point and strain configuration
5. Touch based mesh bed levelling
6. Load experiment execution
7. Data capture
8. Data processing

This sequence of events is repeated for different sensing domains and different loading conditions.

### 6.1 ERT sensor power modes

**Fig. 18:**
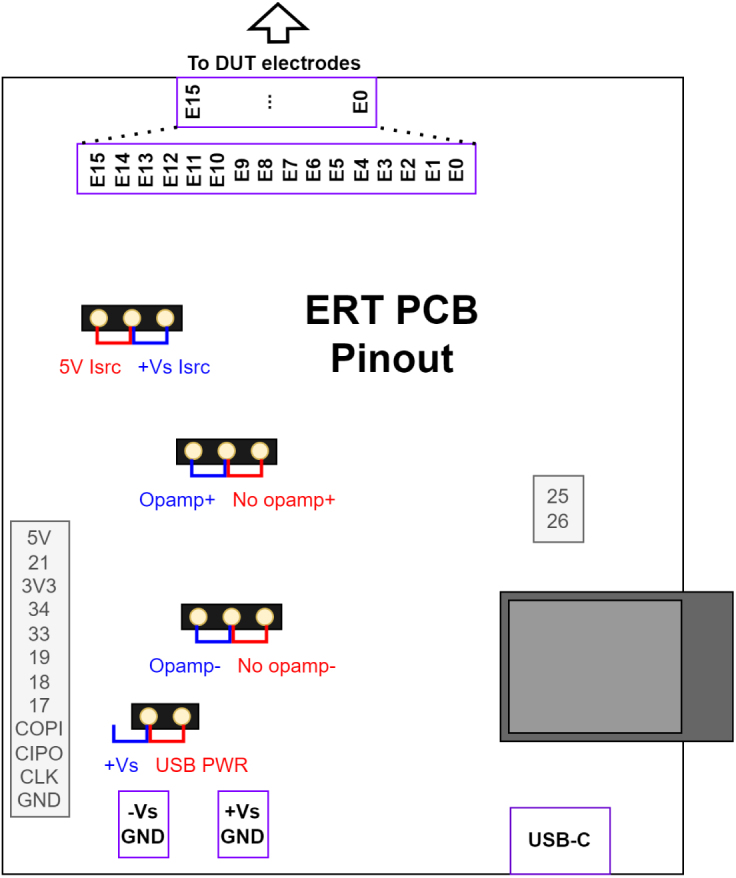
ERT PCB pin-out and power mode options. Blue jumpers are for the bipolar supply mode (i.e. $\pm V_{s}$ attached). Red jumpers are for the ＋5 V USB supply mode. (For interpretation of the references to colour in this figure legend, the reader is referred to the web version of this article.)

Before running any experiments, the ERT sensor power mode must be configured. The jumper configuration for the bipolar supply (blue) and ＋5 V USB single-ended supply (red) mode is shown in Fig. 18.

The bipolar supply first mode is recommended for driving the ERT signal through a wider range of sensing domains at a higher voltage. A bipolar supply of ±20 V is connected to $\pm V_{s}$ for the best performance of the circuit.

The second power mode uses a ＋5 V USB 3.X power supply to run the ERT circuit. The second mode is limited to lower resistance sensing domains that can be tested as it can only drive constant currents using ＋5 V. When using the ＋5 V supply mode the $-V_{s}$ and GND pins on the power input must be shorted for the multiplexers to operate.

For each power mode there will be a distortion of the signal dependent on the power supply voltages and input signal as exemplified in Fig. 15.

### 6.2 ERT sensor programming

The ERT sensor contains an ESP-WROOM32 module [U1] which requires the main_ert.c program to be built and flashed. This can be achieved using the default project template from the ESP-IDF environment [51]. The default ERT circuit firmware main_ert completes the well proven adjacent electrode drive pattern [33], [52], [53], [54]. Upon successful programming of the ERT circuit it will output a constant serial stream of the adjacent electrode pattern ERT data separating each frame of 256 measurements with an ‘A’. The ERT sensor circuit has the capability to send the real-time serial ERT data via a USB serial, Bluetooth, or WiFi connection to a EIT reconstruction capable computer.

### 6.3 ERT sensing domain preparation

For sensing domain fabrication instructions refer to Section 5.2. The ERT sensing domain needs to have sufficiently low adjacent inter-electrode resistance to function, so that the current source will not saturate due the power supply voltage. The ET electrodes can be attached to the domain in many ways as shown in Fig. 6, ensure these connections provide a reliable electrical contact to the sensing domain. Connect sensing domain electrodes to the ERT circuit FPC connector [W1] via an adapter [J8]. The ERT sensing domain must be flat and centred on the sensing domain holder [PR3, PR4] as shown in Fig. 6 (right).

### 6.4 Load application point and strain configuration

Load application points and strains applied to the sensing domain can be configured by altering variables in ertpcb_cfa_reader.py code. A loading sequence consists of a series of load applications, in the form of a strain pulse train, applied to a set of X and Y coordinates on the sensing domain. The main parameters to change for running a loading sequence are given in Table 3.

These can all be found as variables in the software file ertpcb_cfa_reader.py within the main function. Before running this program the serial COM ports may need to be changed in the ertpcb_cfa_reader.py program to match the comports of the CFA and ERT sensor hardware.

**Table 3:** Experimental parameters.

Variable:	Unit:	Description:
Strain speed (v_z_push)	mm/min	The rising/falling edge gradient for each pulse.
Strain limit (strain_limit)	%	The maximum compressive strain allowed.
Load locations (push_points)	[mm, mm]	An array of XY locations of each load pulse.
Reference offset (ref_loc_mm)	[mm, mm]	The XY offset of the zero point of the sensing domain relative to the CFA home reference.

### 6.5 Load experiment execution

Once all hard-coded parameters have been set the command parameters are set and the load experiment begins. To begin the load experiment use the following terminal command:

**Figure:**
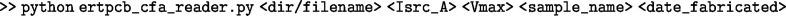

**Figure:**
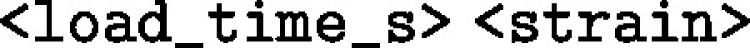

Where <dir/filename> is includes the file directory and file name, <Isrc_A> is the constant current source value set in the ERT circuit in amps, <Vmax> is the maximum allowed voltage to be read by the ADC in volts (e.g. 20 V), <sample_name> is a descriptive sensing domain name,^1^ <date_fabricated> is the sample fabrication date (‘NA’ or leave blank if irrelevant), <load_time_s> is the strain pulse on and off time in seconds, and <strain> is the desired strain applied to the domain as a percentage. If a random test sequence is desired with randomised strain and locations this can be achieved by simply setting the <strain> value to −1.

### 6.6 Touch-based mesh bed levelling

An undulating sensing domain surface can often be present during testing due to an intentionally curved sensor or manufacturing defects. To compensate for an uneven surface a touch-based mesh bed levelling process has been created to improve the quality of the stress/strain data gathered. The process involves the force applicator head travelling towards the sample until a change in force has been detected above 0.1 N. This mitigates the risk of any misalignment with the force applicator surface plane and the sensing domain surface plane and ensures more accurate strain data capture for low strain magnitudes. This touch-based mesh bed levelling is completed before the load sequence experiment begins. The sensing domain and holding trays should not be physically contacted in any form after beginning the ertpcb_cfa_reader.py programme.

### 6.7 Data capture

Once the ertpcb_cfa_reader.py program has completed capturing data, a time-series plot of the 16 inter-electrode resistances, $R_{int}$, will appear. A stable $R_{int}$ is for stable EIT reconstructions of the sensing domain.

Any significant change in the inter-electrode resistance may cause a poor EIT reconstruction result. A significant change in the inter-electrode resistance is defined as a change of more than 2% deviation from the mean and could be a result of, applying force too close to the electrodes themselves, an inherently unstable electrode connection, or an external force applied near the electrode. If the $R_{int}$ values are not stable it will be evident in the plot and there will be a warning message in the console.

Once the inter-electrode resistance plot is closed the program will continue to save all of the data in three separate files for the given filename,

**Fig. 19:**
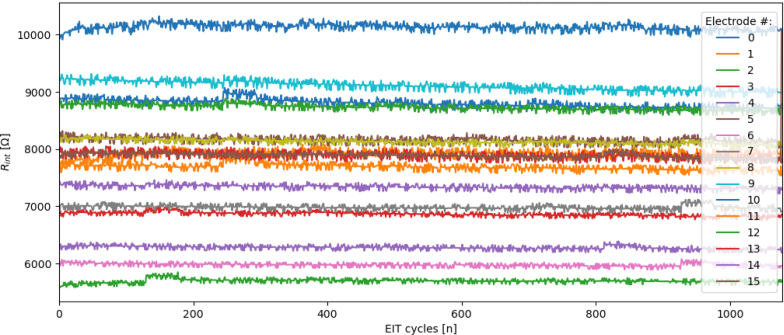
An example plot of the $R_{int}$ values generated on completion of an experiment for a stable experiment. Where the Electrode # ‘i’ represents the resistance between electrode ‘i’ and ‘i + 1’.

1. *filename*.csv - Time series data for ERT voltages, compression forces, and force applicator XYZ locations. The UTC start date and time of the experiment is given in the top row.
2. *filename*.pkl - Logs the same data as the .csv file **and** all of the important experiment parameters into a serialised python ‘pickle’ file.
3. *filename*.gcode - The gcode file of the commands sent to the 3D printer platform for the experiment run.

**Fig. 20:**
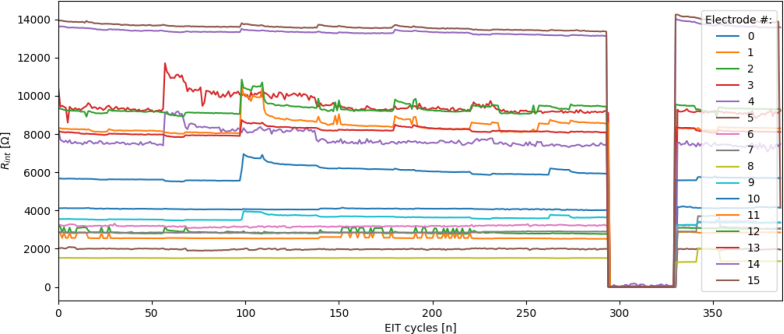
An example plot of the $R_{int}$ values generated on completion of an experiment for an experiment with unstable electrode connections.

An example of an $R_{int}$ plot indicating a stable electrode connection for an experiment is shown in Fig. 19. If there are unstable electrode connection to the domain material a warning will be thrown in the console window and it will usually be evident in the inter-electrode resistance plot given, as exemplified in Fig. 20.

### 6.8 Data processing

Once all of the data has been collected in the above steps, the data can be processed. Data processing includes the following,

1. Pre-processing of the raw voltage, force, and position data. Filtering and data cleaning could be included in this step.
2. Image reconstruction using a chosen EIT algorithm with the pre-processing or raw data. EIT reconstruction could include algorithms such as regularised Newton’s methods [55], neural network based methods [56], [57], and back projection methods [52].
3. Any post-processing of the EIT image reconstruction data and integration with the force applicator stress and/or strain data. Post-processing could include resistance to force inverse modelling, pressure mapping performance metric quantification, an application specific software interface.

In this work EIDORS [58] has been used to complete the EIT reconstructions of the domain and any post processing is then completed with a python program as shown in Section 7.

### 6.9 Construction and operational safety

Various safety concerns must be stated for the construction and operation precautions of this system. This is not a comprehensive safety guide, but will give an overview of some potential safety concerns. Other precautions may be necessary depending on the development location and sensing domain materials used. The construction and operation of the system can each be separated into three parts, the ERT sensor circuit, the CFA, and the sensing domain.

#### 6.9.1 Construction

During the assembly of the ERT sensor circuit the regular health and safety procedures for assembling and soldering a PCB must be followed.

During testing of the CFA as a 3D printer there will be moving parts which could get caught on long hair or collide with a person too close. Steps must be taken to avoid any undesired collisions or any people touching the CFA during operation.

When fabricating the sensing domain often this involves micro/nano sized conductive particles dangerous for inhalation and sometimes dangerous to touch. The material safety datasheet must be consulted for any material used in the sensing domain. Any safety procedures with mixing machines and curing devices must be followed.

#### 6.9.2 Operation

The PCBA can operate on up to ±20 VDC which is within the safe level the SELV as defined by IEC [59]. However, should the contacts of the power supply to the ERT circuit be electrically shorted, a burn or fire hazard may arise. The current on the sensing domain electrodes is limited to prevent a dangerous short circuit current.

During the operation of the CFA there will be moving parts which could collide tangle long hair or collide with the person operating. Steps must be taken to avoid any undesired collisions or any people touching the CFA during operation.

The sensing domain may not be bio-compatible so the material safety datasheet(s) for each domain must be followed for each real world sensor application.

## 7 Validation and characterisation

**Fig. 21:**
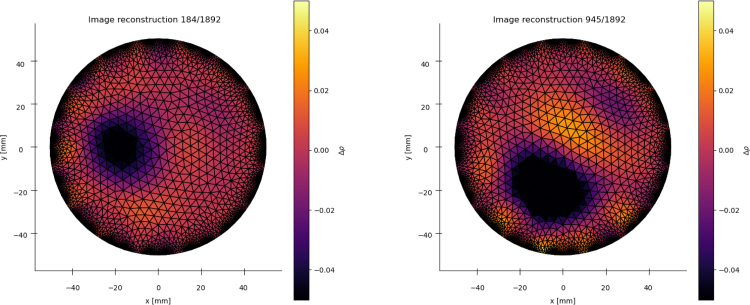
Reconstruction frames from a random push test sequence on a 1 mm thick 100 mm diameter sample. Strain and applied locations (x, y) [mm] - Left: 24% (−14.8, −3.4). Right: 36% (−1.9, −21.3).

**Fig. 22:**
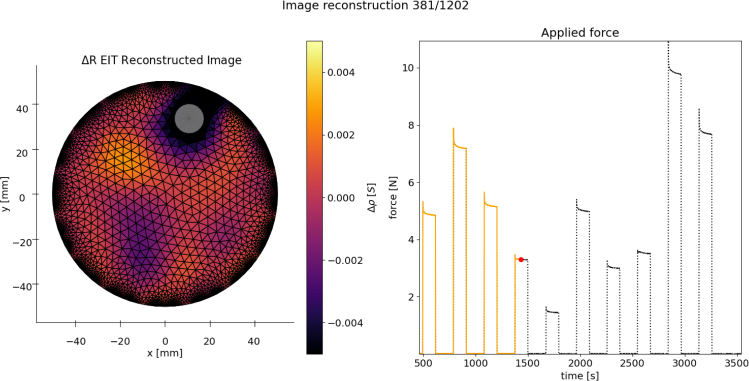
Reconstruction frames from a random push test sequence on a CBSR 100 mm diameter sample. The white circle representing the force applicator location and the red dot on the force plot showing the captured frame in time. (For interpretation of the references to colour in this figure legend, the reader is referred to the web version of this article.)

To show that the system is functional, the plots produced from an EIT reconstruction of the voltage data and the force measurements can be compared for a correlation. Examples are given below in Fig. 21, Fig. 22, showing localised blobs at the known locations of the force applicator.

A raw video of this experiment can be seen in the /examples folder. It can be useful to plot the force profile being applied alongside the EIT reconstruction to verify the data is ready for further processing and modelling.

### 7.1 Sensor capabilities

**Fig. 23:**
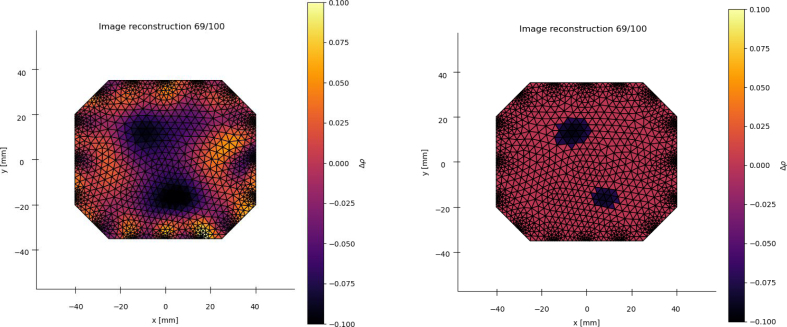
An EIT reconstruction image of a sensing domain with two loads applied simultaneously. Left: Without threshold filtering. Right: With a 75% threshold amplitude filter applied.

Simultaneous application of multiple loads can be achieved with this system using a multi-head force applicator. It has been shown that multiple touch points can be detected as shown in Fig. 23 and in our previous work [24].

**Fig. 24:**
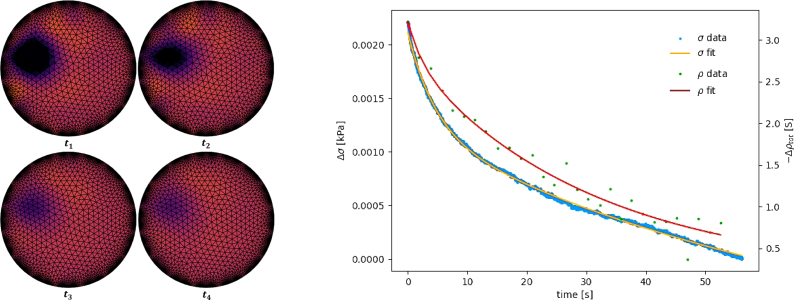
Left: Example sequence a resistive relaxation after a loading event at times $t_{1}$ to $t_{4}$. Right: Example stress, σ, and resistive, ρ, relaxation plot generated from an ERT CFA experiment given a 30% strain step input [19].

A major factor constraining the application of EIT-based sensors is the poor frequency response of the material, which limits the detection of rapid successive loads. The system given in this work allows further research into characterising the transient response of a range of sensing domains in 2D. Examples of how transients have been characterised are shown in Fig. 24

The piezoresistivity of a sensing domain can often vary throughout its volume giving unpredictable results if a homogeneous domain is assumed for the pressure mapping sensor. This system can be used to generate map of the piezoresistivity function of a material surface in 2D dimensions.

## 8 Conclusions

This work has provided the methods and tools to enable further research and development for soft EIT-based pressure sensing systems. The system is low cost, simple to construct, and easy to use. The automation of compression load experiments ensures that experiments are repeatable with quantifiable results and mitigating human error. The automated nature of the CFA device significantly reduces the time to complete a set of experiments and can provide experiment sequences similar to those expected during the real-world application of the sensor. Upon load experiment completion, the system provides clearly formatted raw data files ready for analysis.

Uses of this system vary from 2D piezoresistive material analysis and pressure mapping sensor characterisation. Extensive research has been conducted into one-dimensional (1D) characterisation of piezoresistive materials. However, the characterisation of these materials in two dimensions (2D) has often been overlooked in past literature [34], [43], [60], [61], often due to the complex and invasive methods required. The device can be used to characterise the electromechanical/piezoresistive properties of a soft thick film material in 2D, quantify EIT reconstruction performance, and generate models predicting localised loads from localised resistance changes.

To push the field of EIT-based pressure mapping forward tools are required to standardise testing and reliably acquire quantifiable data for pressure modelling in different sensing domain materials. A toolbox of hardware and software have been described in this work to make EIT-based pressure mapping realisable for more real-world applications.

Proposed future enhancements of the system include minimising noise and offsets in the signal conditioning ERT circuit, adding an auto-calibration procedure to ensure the ADC and $I_{src}$ circuits operate at the expected resolution, and reducing the PCB size. The ERT sensor and Cartesian force applicator system described in this work will help transition this technology into real-world applications.

## CRediT authorship contribution statement

**Richie Ellingham:** Writing – original draft, Visualization, Validation, Software, Project administration, Methodology, Investigation, Formal analysis, Data curation, Conceptualization. **Lui Holder-Pearson:** Writing – review & editing, Supervision. **Chris Pretty:** Writing – review & editing, Supervision. **Tim Giffney:** Writing – review & editing, Supervision, Funding acquisition.

## Declaration of competing interest

The authors declare that they have no known competing financial interests or personal relationships that could have appeared to influence the work reported in this paper.

## Footnotes

1 Please note that the authors have set names which make assumptions about the material properties and the code automatically fills the subsequent sections, see hard coded names in *ert_cfa_reader.py*.
